# Metabolic Modeling of Human Gut Microbiota on a Genome Scale: An Overview

**DOI:** 10.3390/metabo9020022

**Published:** 2019-01-28

**Authors:** Partho Sen, Matej Orešič

**Affiliations:** 1 Turku Centre for Biotechnology, University of Turku and Åbo Akademi University, FI-20520 Turku, Finland; matej.oresic@utu.fi; 2 School of Medical Sciences, Örebro University, 702 81 Örebro, Sweden

**Keywords:** gut microbiome, meta-omics, metagenomics, metabolomics, metabolic reconstructions, genome-scale metabolic modeling, constraint-based modeling, flux balance, host–microbiome, metabolism

## Abstract

There is growing interest in the metabolic interplay between the gut microbiome and host metabolism. Taxonomic and functional profiling of the gut microbiome by next-generation sequencing (NGS) has unveiled substantial richness and diversity. However, the mechanisms underlying interactions between diet, gut microbiome and host metabolism are still poorly understood. Genome-scale metabolic modeling (GSMM) is an emerging approach that has been increasingly applied to infer diet–microbiome, microbe–microbe and host–microbe interactions under physiological conditions. GSMM can, for example, be applied to estimate the metabolic capabilities of microbes in the gut. Here, we discuss how meta-omics datasets such as shotgun metagenomics, can be processed and integrated to develop large-scale, condition-specific, personalized microbiota models in healthy and disease states. Furthermore, we summarize various tools and resources available for metagenomic data processing and GSMM, highlighting the experimental approaches needed to validate the model predictions.

## 1. Introduction

The human gut microbiome consists of trillions of microorganisms such as bacteria, archaea, and unicellular eukaryotes [1,2]. Most gut microbes are facultative obligate anaerobes spanning between five different phyla (Bacteriodetes, Firmicutes, Proteobacteria, Verrumicrobia, and Actinobacteria), with over 1000 species already identified [3]. Several collaborative studies and large consortia such as MetaHIT [4,5], the Human Microbiome Project (HMP) [6,7], and American Gut [8] have taxonomically and functionally profiled the gut microbiome in healthy and various disease states. The composition of the gut microbiota is relatively simple at birth, it undergoes a series of changes in composition, metabolic functions and eventually matures between 3–5 years of age [9]. For any one individual, the composition of the gut microbiome tends to be stable over time. Interestingly, there is a difference in the composition of the gut microbiome within a human population [10,11,12]. Several genetic and environmental factors such as diet, lifestyle, geography, mode of delivery, infection, infant feeding modality (e.g. formula versus breastfed) and medication attribute to these differences, and thereby, shape the gut microbiota during the early stages of life [2,9,13].

The gut microbiome acts as an auxiliary metabolic organ. Several complex carbohydrates, not digested by the host intestinal enzymes, are passed to the microbial community, which are then metabolized in the large intestine [14,15]. The gut microbiota is involved in metabolism of short-chain fatty acid (SCFAs), branched chain fatty acids (BCFAs), branched chain amino acids (BCAAs), biogenic amines, vitamins, bile acids (BAs), and xenobiotics, as well as the production of gases (e.g., CO_2_, CH_4_) [16,17,18]. Gut microbes also affect the host immune system, such as by regulating immune homeostasis versus autoimmunity [19]. Studies in germ-free mice suggest that gut microbiota can induce toll-like receptor (TLR) expression, antigen presenting cells (APCs), and differentiated CD4^+^ T cells [20]. It also maintains the stability of the immune system by providing resistance against pathogens.

Our understanding of the gut microbiome and its role in health and disease has considerably improved with the advent of high-throughput meta-omics technologies. The wealth of data generated by the gut microbiome research, however, begs the development of novel computational tools and mathematical models. Such tools have already enabled researchers to begin exploring complexities of the gut microbiome (Table 1). Several approaches, such as 16S rRNA amplicon sequencing and whole genome shotgun metagenomics sequencing (WGS) have already been used for profiling gut microbes [21]. However, such genome-centric approaches are themselves unable to provide mechanistic insights at the level of individual species, their interactions with other gut flora, and their impact on host metabolism [14,22,23].

**Figure 1:**
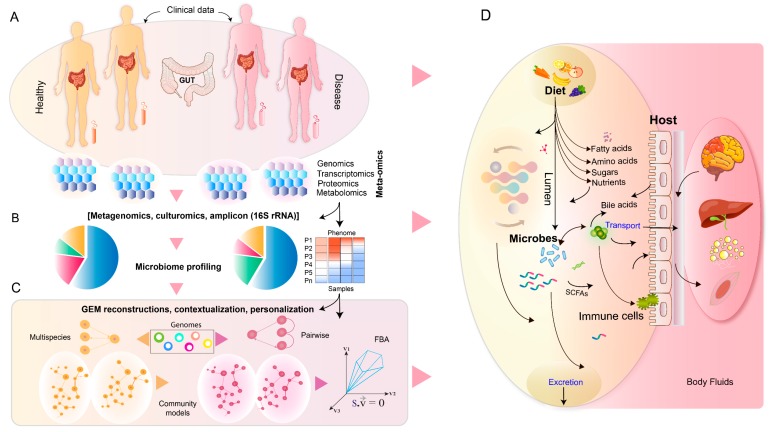
Overview of meta-omics profiling, annotation and genome-scale metabolic reconstructions. (**A**) Fecal, plasma and/or serum samples are taken from healthy and diseased subjects and meta-omics data is generated from these. (**B**) Taxonomic and functional profiling of gut microbes. (**C**) Reconstruction of microbial GEMs. Contextualization and personalization of GEMs with meta-omics datasets. (**D**) Summary of host-microbial interactions in the human gut. GEM simulations to study and understand the intricate relationship among diet, host and microbiota under healthy and disease states.

Genome-scale metabolic modeling (GSMM), a constraint-based mathematical modeling approach has been increasingly used to study gut ecosystems, attempting to elucidate the microbial metabolic interactions with each other and their host [15,24,25,26]. Recently, genome-scale models (GEMs) of catalogued human gut microbes [4,27], based on their metabolic functions, were developed. GEMs can integrate multiple type of biological information within a computational framework [28,29,30,31]. The complex interplay of genes, enzymes, and metabolites provides a scaffold for the integration of multi-omics datasets such as transcriptomics, proteomics, metagenomics, metabolomics and fluxomics (Figure 1). A GEM framework allows researchers to decipher, postulate and test hypotheses linking genotype to phenotype [28,29,30]. Overall, it provides a comprehensive systems biology platform for modeling and analyzing biological systems. 

Herein, we review the role of GSMM in understanding microbial metabolism in the human gut, with a focus on how GEMs have been used to infer diet–microbiome, microbe–microbe and host–microbiome interactions under physiological conditions. We discuss metagenomics profiling, and how meta-omics datasets can be used for building condition-specific personalized community models of gut microbiota. We further summarize the available tools for metagenomic profiling and GSMM. Finally, we highlight and emphasize the experimental techniques and data required to validate the GEM-based predictions. 

## 2. Colonization and Shaping of the Gut Ecosystem 

Early colonization of the gut microbiota in infants is vital for shaping of the intestinal ecosystem at a later age [2,32]. These processes are driven by multiple factors such as mode of delivery, gestational age, maternal diet, environment and host genetics. Additionally, geography, life style, age, certain diseases and drug usage can all affect the gut microbial composition and function [2,33].

The distribution of microbes along the gastrointestinal (GI) tract is non-random, in that, certain species of microbes are co-localizing. *Lactobacillacea*, *Veilonellaceae* and *Helicobacterceae* co-occur in stomach, *Bacillaceae* and *Streptococcaceae* in the small intestine, and *Bacteroidaceae*, *Clostridium*, *Lactobacillaceae* and *Bifidobacterium* in the colon [34]. Dysbiosis in the intestinal ecosystem has been both directly and indirectly linked to autoimmune diseases (e.g., type 1 diabetes (T1D), rheumatoid arthritis (RA)) [35,36], colon cancer [37], type 2 diabetes (T2D) and obesity [5,25], cardiovascular disorders [38], non-alcoholic fatty liver disease (NAFLD) [39,40] as well as inflammatory bowel disease (IDB) [41].

## 3. Gut Microbiome Profiling and Functional Annotation

Metagenomics shotgun sequencing [42] and 16S rRNA amplicon sequencing [43] have been used for profiling gut microbiota from fecal (stool) samples. An appropriately annotated shotgun metagenomics dataset can be used for accurately mapping and predicting microbiota-affected metabolic pathways. These approaches also have proven potential for novel gene discovery [44] and identification of essential functions. Annotation of metagenomics datasets is primarily carried out in two ways: (a) by assembling nucleotide sequences from NGS reads of appropriate length and subsequently predicting the protein coding sequences (called CDS) [45], and (b) by mapping the reads to genome or non-redundant marker gene sets of the relevant organisms guided by the taxonomic profiling [46]. These genes can be clustered, catalogued and aligned against reference database(s) of annotated gene/protein families (e.g., KEGG Orthology [47]), and/or they can be linked to metabolic pathways (e.g., MetaCyc [48]). 

Various computational tools and pipelines have been developed for these sorts of purposes. MOCAT2, for example, provides automated annotation of non-redundant reference catalogues from 18 databases covering various functional categories [45]. HMP Unified Metabolic Analysis Network (HUMAnN2) is a pipeline for profiling the relative abundances of microbes and the activity of their metabolic pathways from metagenomics data [46,49]. MEtaGenome ANalyzer (MEGAN) is an interactive and comprehensive microbiome analysis toolbox, that allows researchers to explore and analyze large-scale metagenomics datasets both from taxonomic and functional perspectives [50]. Metagenomics Rast (MG-RAST), is a RAST (Rapid Annotation using Subsystem Technology) server for automated annotation of metagenomics datasets [51]. Integrated Microbial Genomes & Microbiomes (IMG/M) is another server-based system that supports the annotation and analysis of microbiome datasets [52]. There is a plethora of tools for sequence assembly, gene prediction and phylogenetic classification which underpin many of these processes, and these tools are extensively reviewed elsewhere [53].

Functional annotation of metagenomics datasets poses several challenges in itself [53,54]. Although metagenomics data categorizes microbial functions at the community level, it fails to suggest a mechanistic explanation for how these functions arise. To understand the intricate relationship between microbial components, such as genes, proteins and metabolites, and their influence on host metabolism via different biochemical pathways, microbe-specific metabolic models need to be developed at the genome scale.

## 4. A Constraint-Based Strategy and Tools for Genome-Scale Metabolic Modeling of Gut Microbiota

A rapid increase in use of shotgun metagenomics, the availability of model organisms, and the number of meta-omics datasets in public repositories, gives an opportunity to develop metabolic reconstructions of human gut microbes. These reconstructions can be converted into quantitative mathematical models that can be used to study metabolism at the genome scale [28,55,56,57,58]. Current tools and resources for gut microbiome modeling are listed in Table 1.

In a GEM, uptake or secretion of certain metabolites over time (denoted as their ‘flux’), enzymes/transcript abundances and ON/OFF gene expression can be constrained using information from datasets generated by quantitative fluxomic, metabolomic, transcriptomic and proteomic experiments. By applying these constraints, GEMs can be contextualized to a particular state or condition. These condition-specific/contextualized models can provide information about the activity of metabolic pathways, metabolite flux, cellular growth, and provide estimates of the overall metabolic capacities of these gut microbes. GSMM use FBA [28], a constraint-based approach (CBA), to predict organisms’ phenotypes [28]. A tutorial on linear programming and FBA is available in [28].

GSMM has been applied to study gut microbial metabolism and its interactions with the host. Recently, AGORA (Assembly of Gut Organisms through Reconstruction and Analysis) was published, which carried out semi-automatic metabolic reconstruction of 773 human gut bacteria (205 genera, 605 species) [26]. The authors modeled metabolic interactions among microbial species based on their metabolic potential and availability of nutrients. This approach has identified and defined growth medium for *Bacteroides caccae ATCC 34185*. Moreover, these metabolic reconstructions have been used to infer metabolic diversity of microbial communities. The AGORA framework can be coupled with, for example Recon 2, a generic reconstruction of human metabolism, which in turn can be used to study host–microbiome interactions. AGORA reconstructions are publicly available via the Virtual Metabolic Human (VMH) [74] database (https://vmh.life/). In addition, BiGG Models [73] (http://bigg.ucsd.edu/) and the Human Metabolic Atlas [76] (http://metabolicatlas.org/) are other open access knowledge bases for metabolic reconstructions. 

Kbase [63] (https://kbase.us/) and ModelSEED [75] (http://modelseed.org/) are the web-based servers for automatic reconstruction of microbial GEMs by integrating genome sequences and/or metagenomics datasets. The COnstraint-Based Reconstruction and Analysis (COBRA) [59,60,61] and RAVEN (Reconstruction, Analysis, and Visualization of Metabolic Networks) [62] toolboxes are stand-alone MATLAB software suites with collections of basic and advanced functions for genome-scale reconstructions and modeling. The Microbiome Modeling Toolbox [82] extends the functionality of the COBRA toolbox to use metagenomic data for modeling microbe–microbe/host–microbe metabolic interactions and modeling personalized microbial communities. Draft GEMs generated by these platforms are then curated for the occurrence of genes, metabolites, reactions and their associations based on evidence from the literature and expert knowledge of metabolism. Quality control checks, which are performed to eliminate false positives, also enhance the predictability of GEMs [55]. 

## 5. Reconstruction of Condition-Specific Personalized Gut Microbiota Models 

In a metabolic model, numerous genes and metabolites are associated by way of metabolic pathways deemed to be thermodynamically feasible. These models are formalized and applied over the entire microbiota community model [82]. Various efforts have already been made to integrate metagenomic data with a genome-scale framework [26,83]. However, approaches to integrate other kinds of meta-omics data are still in the early phases of development. 

Shotgun metagenomics and 16S rRNA data have guided the selection of representative microbes (species or strains) in a community [24]. Integration of meta-omics datasets such as metatranscriptomics, metaproteomics together with fecal metabolomics with the microbiota metabolic modeling framework can constrain the model, improving the accuracy of its representation of the biological system. Moreover, meta-omics data can be applied to develop condition-specific microbiota models (Figure 1) such as metabolic reconstruction of gut microbiota in lean vs. obese subjects. Likewise, a microbiota model can be personalized for an individual subject by combining the metagenomics information with other phenomics datasets. Metagenomics, metatranscriptomics and metaproteomics data can provide an estimate for enzymatic and pathway activities in the gut [49], which approximate the metabolic activity in the gut of an individual under specified conditions. 

Context-based, personalized microbiota models have already been used to study various conditions [28,55,56,61,84]. An array of analysis can be performed with these models. Flux Variability Analysis (FVA) [28,85] can estimate the maximal and minimal possible flux differences (flux span) for a specific metabolic exchange reaction of a specific microbial strain, pair of strains, or community as a whole. It determines the potential of a reaction to carry out flux under the applied constraints/conditions. FVA can thus be used to compute strain-specific exchange fluxes for a particular metabolite that can be compared with the net metabolite exchanges in the community. Moreover, it can evaluate the role of individual microbe for metabolite production. On the other hand, shadow price (SP) of a metabolite determines whether it is limiting for an optimal objective function (growth or biomass production) [28,61]. A negative SP suggests that flux through the objective function would increase with the increase in the concentration of the metabolite. As an example, SP analysis has already identified several microbial strains that decrease ursodeoxycholate (UDCA) biosynthesis by limiting its precursors [83]. 

Food metabolomics datasets detailing dietary constituents have been used to constrain the nutrient uptake rates of microbiota models [58]. Diet acts as a ‘spooning media’ for the microbiome. Several diets such as a typical Western diet, high fiber diet [26], average European diet [26], breast milk [58], and Ready-to-Use Therapeutic Foods (RUTFs) [24], have been designed. The diet designer tool included as part of the aforementioned [74] can be used to calculate range of dietary fluxes, given the metabolite concentrations. On the other hand, fecal, serum and plasma metabolomics data can be used to confirm the identity of microbial metabolites produced by the models [24,25]. 

## 6. Modeling the Effect of Diet on Gut Microbiome

Diet is the direct regulator of microbial metabolism in the gut ecosystem; dietary patterns have profound effect on gut colonization and the shaping of the gut microbiome during the early stages of life [9]. Western diets are associated with a *Bacteroides* enterotype whereas plant-based polysaccharides are associated with a *Prevotella* enterotype [86]. Mostly, three primary macronutrients such carbohydrates, proteins, and fats are known to affect the gut microbial composition [18]. 

GSMM has already begun to be used to help improve mechanistic understanding of gut microbial metabolism and its dietary interactions [24,25,26]. Computational tools such as COMET [65], BacArena [64], dOptCom [68], MatNet [87], DyMMM [67], MCM [66], and CASINO [25] were designed to study diet–microbiome interactions. CASINO was able to predict the interactions along the diet-microbiota-host axis in 45 obese and overweight individuals [25]. Furthermore, this study estimated the metabolic capabilities of microbes in the lumen of obese and overweight individuals. The model predicted a significant change in the amino acids and SCFAs levels in response to dietary intervention. The model predictions were further validated by fecal and blood metabolomics data. In another study, GSMM was used to predict and elucidate the underlying interactions between *Bacteroides thetaiotamicron*, *Eubacterium rectale* and *Methanobrevibacter smithii*, when subjected to different gut ecosystems [15,22]. Recently, GEM-based predictions were used to evaluate the effect of RUTFs on gut microbiome of healthy and malnourished children from Bangladesh and Malawi [24]. This methodology can be further extended to study the effect of health supplements, prebiotics and probiotics on the human gut microbiota.

## 7. Multispecies Modeling and Interactions in the Gut Community 

Microbial species or strains with high abundances in samples are often selected for pairwise or community modeling [24,26]. Two or more microbial GEMs are joined together along their extracellular compartments to build a community model [82]. The community model is linked to a “common compartment” mimicking the human gut, through which exchange of metabolites takes place. A community biomass, i.e., the sum of biomasses estimated for each microbe, and coupling constraints are added [82].

Pairwise analysis of microbes in the community has determined their metabolic relationships when introduced to different types of diets [24,26,83]. However, in vitro screening of microbial pairs can be laborious and expensive. When subjected to Western and high fiber diets under aerobic and anaerobic conditions, pairwise modeling has predicted six different interactions between gut microbes such as competition, parasitism, amensalism, neutralism, commensalism and mutualism [26]. Furthermore, pairwise models developed from personalized gut microbiomes have been interrogated for single, cooperative, and community-wide bile acid production potential [83]. This strategy has identified several microbe pairs producing secondary BAs. For instance, *Bacteroides spp.* and *R. gnavus* can cooperatively produce UDCA [83]. In another study, the rate of butyrate production increased by pairs of microbes as compared to a single species, when studied in the gut communities of healthy Bangladeshi and Malawian children [24].

Alternatively, correlation-based co-occurrence topological networks looking at abundant metagenomic species can be developed [88,89]. Such a network can predict positive or negative associations between the microbes. Microbe–microbe co-occurrence pairs of interest can be selected and evaluated by in vitro co-culture experiments [90]. Interestingly, co-occurring species compete strongly for metabolic resources, which are required for cellular growth and maintenance. In this context, the network analysis can be extended to incorporate different metrics such as competition and complementarity indices, which can be used to further characterize/quantify the degree of metabolic interactions between the selected pairs of microbes.

## 8. Metabolic Modeling of Host–Microbiome Interactions

Gut microbiota can harvest nutrients and energy from the diet. During these processes, small molecules (metabolites) are produced. Some of these metabolites can be beneficial for host and microbial symbionts [16,18,84]. One such metabolite is butyrate, a bacterial fermentation product that fuels the colonic epithelium [22]. In fact, butyrate is the primary energy source for colonocytes. In mammals, the production of cresols from tyrosine have been linked to various species of *Clostridium*, *Bifidobacterium*, and *Bacteroides*, and altered 4-cresol levels in human urine have been associated with weight loss in IBD [17]. The primary conjugated BAs produced by liver are deconjugated and biotransformed by gut microbes, affecting host signaling and metabolism [83]. Also, BAs can activate the innate immune genes which in turns alters the gut microbial composition. It also inhibits the growth of pathogens in the gut. 

GEMs have been expanded to study metabolism in humans. Human generic metabolic reconstructions such as Recon 1 [91] and the Edinburgh Human Metabolic Network (EHMN) [92] were developed with a vision to integrate and analyze biological datasets. Similarly, Recon 2 [56,93] and Recon 3D [94], and Human Metabolic Reaction (HMR) [95,96], were designed, that comprehensively captured human metabolism. A metabolic reconstruction of human small intestinal epithelial cells (sIECs) was assembled and manually curated [97]. sIECs were used to study the physiological functionality of the small intestine and their overall role in human metabolism. These models incorporate transporters present in the human gut [94,97,98], while some of them are putatively identified. Furthermore, several functional cell or tissue-specific GEMs have been generated for the liver [96], brain [99], adipocytes [95] and myocytes [100], using semi-automated approaches [101]. In addition, a gender-specific, whole-body metabolism (WBM) reconstruction was developed to capture and characterize the metabolism of 20 human organs [102]. A WBM framework can be constrained with dietary, physiological parameters and omics datasets. Such a framework was used to link organ-level metabolic processes in 149 subjects induced by their gut microbiota.

The Microbiome Modeling Toolbox [82], deployed under the COBRA suite, includes several functions for modeling complex metabolic interactions between the host and gut microbiota. It can integrate microbe (AGORA [26], BiGG [73]) and host (Recon [56,91,94]) metabolic reconstructions. Similarly, a common compartment mimicking the human gut is added, which enables pooling and exchange of metabolites between the microbes, lumen and the host cells.

In a different context, the microbiome-induced immune response is currently well established. An imbalance in gut microbial composition has been linked to inflammatory and autoimmune diseases [103,104,105,106]. Various immune cells including CD4^+^ effector T cells (particularly Th1, Th2, Th17 and iTreg), CD8^+^ T cells (cytotoxic) and macrophages undergo metabolic reprogramming during proliferation and differentiation processes [107]. The macrophage (RAW 264.7 cell line) model was developed to study immunoactivation and immunosuppression [108]. Metabolic reconstructions of immune cells are currently unavailable. By developing GEMs for host immune cells [57], might guide us to study, the microbiome-mediated immunometabolic responses under various health/disease conditions.

## 9. Model Predictions and Experimental Validation 

To establish the biological relevance of metabolic models, the congruence between model predictions and experimental data is of utmost importance. GEM-based predictions can be validated by existing data, knowledge and bibliographical evidence. For instance, metabolites secreted by gut microbiota can be compared with the concentrations of metabolites found in fecal and blood samples [24,25]. Furthermore, blood metabolomics data can be used for validation of metabolites predicted as being transported across the human gut. Meta-omics datasets [109] can be used to estimate the abundances of gut enzymes and microbial pathways for an individual species or strain [49]. The pathway abundances can be compared with the enrichment and usage (flux) of GEM-predicted pathway(s). GSMM can be applied to quantify dietary nutrient uptake of gut microbes and their metabolic interactions with the host. To understand the regulation of host metabolism by gut microbes, germ-free (GF) and conventionally raised (CONV-R) mice are usually used [110]. These mice can be raised on different diets and then euthanized, with samples analyzed by meta-omics analyses. The generated datasets can be used for contextualization and validation of GEMs. Furthermore, the theoretical growth rate of a microbe can be validated by culturing species in a specific media [25,26]. In addition, the predicted metabolic interactions between microbes, regulation of co-occurrence network, and dietary cross-feeding can be validated by mono- and co-culture experiments [90].

## 10. Concluding Remarks and Future Perspectives 

Integration of meta-omics datasets and genome-wide metabolic reconstructions provide a framework for interrogating and suggesting mechanistic workings of diet-microbe-host metabolic interaction. However, such integrative methods are still evolving and require extensive and robust experimental validation. 

Profiling and culturing gut microbes at the strain level, under controlled conditions, remains challenging. Recently, an integrated approach involving targeted phenotypic culturing, WGS, phylogenetic analysis and computational modeling has succeeded in culturing a substantial portion of bacteria previously declared to be ‘unculturable’ under laboratory conditions. This approach identified 137 bacterial species, including novel species isolated from pure cultures [111]. Furthermore, the culturomics techniques are currently used for filling the gap by isolating the unknown or novel members of the gut community [111,112]. 

In studies of gut microbial communities, there is increasing interest in mechanistic approaches, in contrast to solely genome-centric approaches. Correspondingly, GSMM is widely used as a preferred computational method for studying gut microbial metabolism and its interaction with the host. Additionally, GEMs can be contextualized and personalized using longitudinal meta-omics datasets, providing a snapshot of metabolic processes over time. Personalized microbiota models may help to reduce the costs of clinical studies, predict markers and contribute to the development of potential treatments at either the individual patient level, or for a defined patient group [83,113]. Many efforts are ongoing, aiming to couple pharmacokinetic and constraint-based models to study drug-microbe-diet interactions [114]. However, a limitation of GSMM approach is that GEMs are stoichiometric models, and cannot, in their current form at least, incorporate metabolite concentrations or enzyme kinetics (V_max_, K_m_, K_cat_) [115,116]. Albeit more limited in scope, kinetic modeling [116] may help improve understanding of the dynamics of metabolic pathways in the human gut.

As indicated in this review, GSMM and CBA have provided computational tools and frameworks to study metabolism of gut microbiota. These tools guided researchers to study and identify the metabolic functions of individual microbes in the gut community. It also helped to infer their spatial dynamics, environmental interactions and metabolic resource allocations under a certain condition. We believe that, a combination of several computational and experimental approaches, may reveal the complex and diverse structure of the human gut microbiome and its underlying interactions with the host metabolic machinery. It might bridge the gaps in gut microbiome research and thereby, enhance our knowledge of human gut microbiota under health/disease conditions.

## Figures and Tables

**Table 1:** Tools and resources for genome-scale metabolic modeling.

Toolboxes	Short Description	Source or Reference
	**Modeling Tools**	
COBRA (Microbiome Modeling Toolbox)	A MATLAB suite for constraint-based modeling (CBM), includes tools and methods for pairwise and community modeling of microbiota. COBRA can be used for GEM reconstruction and analysis.	[59,60,61]
RAVEN (CASINO)	A MATLAB suite for CBM, includes tools for modeling diet-microbiota interactions. It can be used for GEM reconstruction and analysis.	[62]
Kbase	A web-based tool for systems biology and metabolic modeling. It can be used for automatic GEM reconstruction and analysis.	[63]
BacArena	An R-package for individual-based and CBM of microbes in a gut community.	[64]
COMETS	A software platform for stoichiometric modeling of individual microbial species using dynamic flux balance analysis (FBA).	[65]
MCM	A tool for CBM of microbial community model, based on conventional FBA.	[66]
DyMMM	A tool for CBM that integrates multiple microbial species into a dynamic community model.	[67]
OptCom	A modeling framework to perform FBA of microbial communities.	[68]
SteadyCom	A toolbox that can be used to predict the changes in microbial species abundance in response to the dietary changes.	[69]
MetExplore	An open access web-server for integrative analysis of metabolomic datasets and genome-scale metabolic networks.	[70]
MMinte	An integrated pipeline for modeling the pairwise interactions within a microbial network.	[71]
jQMM library	An open-source, Python-based framework for modeling internal metabolic fluxes. The toolbox can be used for FBA and 13C Metabolic Flux Analysis (MFA).	[72]
	**Model repositories and databases**	
BiGG database	An open access database for gold standard GEMs.	[73]
Virtual Metabolic Human (VMH)	An open access database for human and gut microbial metabolism (GEMs).	[74]
ModelSEED	A web-based resource for metabolic modeling.	[75]
Human Metabolic Atlas (HMA)	An open access web-based resource for human metabolism.	[76]
	**Metabolic Pathways and Enzyme databases**	
MetaCyc/HumanCyc	A curated database of experimentally validated metabolic pathways. HumanCyc is a database of curated human metabolic pathways.	[48]
KEGG	A resource comprised of databases including large-scale molecular datasets and detailed pathway information.	[77,78]
BRENDA	An information retrieval system focusing on enzymes and their ligands.	[79]
REACTOME	An open access database of biological pathways.	[80]
UniProt.	An open access database of curated protein information.	[81]

## Abbreviations

NGS	Next-Generation Sequencing
GSMM	Genome-Scale Metabolic Modeling
HMP	Human Microbiome Project
SCFAs	Short-Chain Fatty Acids
BCFAs	Branched Chain Fatty Acids
BCAAs	Branched Chain Amino Acids
BAs	Bile Acids
TLR	Toll-Like Receptor
APCs	Antigen Presenting Cells
WGS	Whole Genomes Shotgun metagenomics sequencing
GEMs	Genome-Scale Models
T1D	Type 1 Diabetes
RA	Rheumatoid Arthritis
T2D	Type 2 Diabetes
NAFLD	Non-Alcoholic Fatty Liver Disease
IDB	Bowel Disease
CDS	Protein Coding Sequences
KEGG	Kyoto Encyclopedia of Genes and Genomes
HUMAnN2	HMP Unified Metabolic Analysis Network
MEGAN	MEtaGenome ANalyzer
MG-RAST	Metagenomics Rast
RAST	Rapid Annotation using Subsystem Technology
IMG/M	Integrated Microbial Genomes and Microbiomes
FBA	Flux Balance Analysis
CBA	Constraint-Based Approach
AGORA	Assembly of Gut Organisms through Reconstruction and Analysis
VMH	Virtual Metabolic Human
COBRA	COnstraint-Based Reconstruction and Analysis
RAVEN	Reconstruction, Analysis, and Visualization of Metabolic Networks
FVA	Flux Variability Analysis
SP	Shadow Price
UDCA	Ursodeoxycholate
RUTFs	Ready-to-Use Therapeutic Foods
EHMN	Edinburgh Human Metabolic Network
HMR	Human Metabolic Reaction
sIECs	small Intestinal Epithelial Cells
WBM	Whole-Body Metabolism
GF	Germ-Free
CONV-R	Conventionally Raised

## References

[1] Thursby E., Juge N. (2017). Introduction to the human gut microbiota. Biochem. J..

[2] Schmidt T.S.B., Raes J., Bork P. (2018). The Human Gut Microbiome: From Association to Modulation. Cell.

[3] Hugon P., Dufour J.C., Colson P., Fournier P.E., Sallah K., Raoult D. (2015). A comprehensive repertoire of prokaryotic species identified in human beings. Lancet Infect. Dis..

[4] Li J., Jia H., Cai X., Zhong H., Feng Q., Sunagawa S., Arumugam M., Kultima J.R., Prifti E., Nielsen T. (2014). An integrated catalog of reference genes in the human gut microbiome. Nat. Biotechnol..

[5] Pedersen H.K., Gudmundsdottir V., Nielsen H.B., Hyotylainen T., Nielsen T., Jensen B.A., Forslund K., Hildebrand F., Prifti E., Falony G. (2016). Human gut microbes impact host serum metabolome and insulin sensitivity. Nature.

[6] Gevers D., Knight R., Petrosino J.F., Huang K., McGuire A.L., Birren B.W., Nelson K.E., White O., Methe B.A., Huttenhower C. (2012). The Human Microbiome Project: A community resource for the healthy human microbiome. PLoS Biol..

[7] Integrative H. (2014). The Integrative Human Microbiome Project: Dynamic analysis of microbiome-host omics profiles during periods of human health and disease. Cell Host Microbe.

[8] McDonald D., Hyde E., Debelius J.W., Morton J.T., Gonzalez A., Ackermann G., Aksenov A.A., Behsaz B., Brennan C., Chen Y. (2018). American Gut: An Open Platform for Citizen Science Microbiome Research. mSystems.

[9] Rodriguez J.M., Murphy K., Stanton C., Ross R.P., Kober O.I., Juge N., Avershina E., Rudi K., Narbad A., Jenmalm M.C. (2015). The composition of the gut microbiota throughout life, with an emphasis on early life. Microb. Ecol. Health Dis..

[10] Faith J.J., Guruge J.L., Charbonneau M., Subramanian S., Seedorf H., Goodman A.L., Clemente J.C., Knight R., Heath A.C., Leibel R.L. (2013). The long-term stability of the human gut microbiota. Science.

[11] Costea P.I., Coelho L.P., Sunagawa S., Munch R., Huerta-Cepas J., Forslund K., Hildebrand F., Kushugulova A., Zeller G., Bork P. (2017). Subspecies in the global human gut microbiome. Mol. Syst. Biol..

[12] Hisada T., Endoh K., Kuriki K. (2015). Inter- and intra-individual variations in seasonal and daily stabilities of the human gut microbiota in Japanese. Arch. Microbiol..

[13] Wen L., Duffy A. (2017). Factors influencing the gut microbiota, inflammation, and type 2 diabetes. J. Nutr..

[14] Ji B., Nielsen J. (2015). New insight into the gut microbiome through metagenomics. Adv. Genom. Genet..

[15] Shoaie S., Karlsson F., Mardinoglu A., Nookaew I., Bordel S., Nielsen J. (2013). Understanding the interactions between bacteria in the human gut through metabolic modeling. Sci. Rep..

[16] Lamichhane S., Sen P., Dickens A.M., Oresic M., Bertram H.C. (2018). Gut metabolome meets microbiome: A methodological perspective to understand the relationship between host and microbe. Methods.

[17] Nicholson J.K., Holmes E., Kinross J., Burcelin R., Gibson G., Jia W., Pettersson S. (2012). Host-gut microbiota metabolic interactions. Science.

[18] Rowland I., Gibson G., Heinken A., Scott K., Swann J., Thiele I., Tuohy K. (2018). Gut microbiota functions: Metabolism of nutrients and other food components. Eur. J. Nutr..

[19] Molloy M.J., Bouladoux N., Belkaid Y. (2012). Intestinal microbiota: Shaping local and systemic immune responses. Semin. Immunol..

[20] Valentini M., Piermattei A., Di Sante G., Migliara G., Delogu G., Ria F. (2014). Immunomodulation by gut microbiota: Role of Toll-like receptor expressed by T cells. J. Immunol. Res..

[21] Hamady M., Knight R. (2009). Microbial community profiling for human microbiome projects: Tools, techniques, and challenges. Genome Res..

[22] Shoaie S., Nielsen J. (2014). Elucidating the interactions between the human gut microbiota and its host through metabolic modeling. Front. Genet..

[23] Bauer E., Thiele I. (2018). From Network Analysis to Functional Metabolic Modeling of the Human Gut Microbiota. mSystems.

[24] Kumar M., Ji B., Babaei P., Das P., Lappa D., Ramakrishnan G., Fox T.E., Haque R., Petri W.A., Bäckhed F. (2018). Gut microbiota dysbiosis is associated with malnutrition and reduced plasma amino acid levels: Lessons from genome-scale metabolic modeling. Metab. Eng..

[25] Shoaie S., Ghaffari P., Kovatcheva-Datchary P., Mardinoglu A., Sen P., Pujos-Guillot E., de Wouters T., Juste C., Rizkalla S., Chilloux J. (2015). Quantifying diet-induced metabolic changes of the human gut microbiome. Cell Metab..

[26] Magnúsdóttir S., Heinken A., Kutt L., Ravcheev D.A., Bauer E., Noronha A., Greenhalgh K., Jäger C., Baginska J., Wilmes P. (2017). Generation of genome-scale metabolic reconstructions for 773 members of the human gut microbiota. Nat. Biotechnol..

[27] Qin J., Li R., Raes J., Arumugam M., Burgdorf K.S., Manichanh C., Nielsen T., Pons N., Levenez F., Yamada T. (2010). A human gut microbial gene catalog established by metagenomic sequencing. Nature.

[28] Orth J.D., Thiele I., Palsson B.Ø. (2010). What is flux balance analysis?. Nat. Biotechnol..

[29] Price N.D., Reed J.L., Palsson B.Ø. (2004). Genome-scale models of microbial cells: Evaluating the consequences of constraints. Nat. Rev. Microbiol..

[30] O’Brien E.J., Monk J.M., Palsson B.O. (2015). Using genome-scale models to predict biological capabilities. Cell.

[31] Bordbar A., Monk J.M., King Z.A., Palsson B.O. (2014). Constraint-based models predict metabolic and associated cellular functions. Nat. Rev. Genet..

[32] Backhed F., Roswall J., Peng Y., Feng Q., Jia H., Kovatcheva-Datchary P., Li Y., Xia Y., Xie H., Zhong H. (2015). Dynamics and Stabilization of the Human Gut Microbiome during the First Year of Life. Cell Host Microbe.

[33] Milani C., Duranti S., Bottacini F., Casey E., Turroni F., Mahony J., Belzer C., Delgado Palacio S., Arboleya Montes S., Mancabelli L. (2017). The First Microbial Colonizers of the Human Gut: Composition, Activities, and Health Implications of the Infant Gut Microbiota. Microbiol. Mol. Biol. Rev..

[34] Zhang Z., Geng J., Tang X., Fan H., Xu J., Wen X., Ma Z.S., Shi P. (2014). Spatial heterogeneity and co-occurrence patterns of human mucosal-associated intestinal microbiota. ISME J..

[35] Boerner B.P., Sarvetnick N.E. (2011). Type 1 diabetes: Role of intestinal microbiome in humans and mice. Ann. N. Y. Acad. Sci..

[36] Abdollahi-Roodsaz S., Abramson S.B., Scher J.U. (2016). The metabolic role of the gut microbiota in health and rheumatic disease: Mechanisms and interventions. Nat. Rev. Rheumatol..

[37] Sears C.L., Garrett W.S. (2014). Microbes, microbiota, and colon cancer. Cell Host Microbe.

[38] Jonsson A.L., Backhed F. (2017). Role of gut microbiota in atherosclerosis. Nat. Rev. Cardiol..

[39] Spencer M.D., Hamp T.J., Reid R.W., Fischer L.M., Zeisel S.H., Fodor A.A. (2011). Association between composition of the human gastrointestinal microbiome and development of fatty liver with choline deficiency. Gastroenterology.

[40] He X., Ji G., Jia W., Li H. (2016). Gut Microbiota and Nonalcoholic Fatty Liver Disease: Insights on Mechanism and Application of Metabolomics. Int. J. Mol. Sci..

[41] Wlodarska M., Kostic A.D., Xavier R.J. (2015). An integrative view of microbiome-host interactions in inflammatory bowel diseases. Cell Host Microbe.

[42] Simon C., Daniel R. (2011). Metagenomic analyses: Past and future trends. Appl. Environ. Microbiol..

[43] Carlos N., Tang Y.W., Pei Z. (2012). Pearls and pitfalls of genomics-based microbiome analysis. Emerg. Microbes Infect..

[44] Sharma V.K., Kumar N., Prakash T., Taylor T.D. (2010). MetaBioME: A database to explore commercially useful enzymes in metagenomic datasets. Nucleic Acids Res..

[45] Kultima J.R., Coelho L.P., Forslund K., Huerta-Cepas J., Li S.S., Driessen M., Voigt A.Y., Zeller G., Sunagawa S., Bork P. (2016). MOCAT2: A metagenomic assembly, annotation and profiling framework. Bioinformatics.

[46] Abubucker S., Segata N., Goll J., Schubert A.M., Izard J., Cantarel B.L., Rodriguez-Mueller B., Zucker J., Thiagarajan M., Henrissat B. (2012). Metabolic reconstruction for metagenomic data and its application to the human microbiome. PLoS Comput. Biol..

[47] Kanehisa M., Sato Y., Kawashima M., Furumichi M., Tanabe M. (2015). KEGG as a reference resource for gene and protein annotation. Nucleic Acids Res..

[48] Caspi R., Billington R., Ferrer L., Foerster H., Fulcher C.A., Keseler I.M., Kothari A., Krummenacker M., Latendresse M., Mueller L.A. (2016). The MetaCyc database of metabolic pathways and enzymes and the BioCyc collection of pathway/genome databases. Nucleic Acids Res..

[49] Franzosa E.A., McIver L.J., Rahnavard G., Thompson L.R., Schirmer M., Weingart G., Lipson K.S., Knight R., Caporaso J.G., Segata N. (2018). Species-level functional profiling of metagenomes and metatranscriptomes. Nat. Methods.

[50] Huson D.H., Auch A.F., Qi J., Schuster S.C. (2007). MEGAN analysis of metagenomic data. Genome Res..

[51] Glass E.M., Wilkening J., Wilke A., Antonopoulos D., Meyer F. (2010). Using the metagenomics RAST server (MG-RAST) for analyzing shotgun metagenomes. Cold Spring Harbor Protocols.

[52] Chen I.A., Markowitz V.M., Chu K., Palaniappan K., Szeto E., Pillay M., Ratner A., Huang J., Andersen E., Huntemann M. (2017). IMG/M: Integrated genome and metagenome comparative data analysis system. Nucleic Acids Res..

[53] Prakash T., Taylor T.D. (2012). Functional assignment of metagenomic data: Challenges and applications. Brief. Bioinform..

[54] Gilbert J.A., Field D., Swift P., Thomas S., Cummings D., Temperton B., Weynberg K., Huse S., Hughes M., Joint I. (2010). The taxonomic and functional diversity of microbes at a temperate coastal site: A ‘multi-omic’study of seasonal and diel temporal variation. PLoS ONE.

[55] Thiele I., Palsson B.Ø. (2010). A protocol for generating a high-quality genome-scale metabolic reconstruction. Nat. Protocols.

[56] Thiele I., Swainston N., Fleming R.M., Hoppe A., Sahoo S., Aurich M.K., Haraldsdottir H., Mo M.L., Rolfsson O., Stobbe M.D. (2013). A community-driven global reconstruction of human metabolism. Nat. Biotechnol..

[57] Sen P., Kemppainen E., Orešič M. (2018). Perspectives on Systems Modeling of Human Peripheral Blood Mononuclear Cells. Front. Mol. Biosci..

[58] Sen P., Mardinogulu A., Nielsen J. (2017). Selection of complementary foods based on optimal nutritional values. Sci. Rep..

[59] Becker S.A., Feist A.M., Mo M.L., Hannum G., Palsson B.O., Herrgard M.J. (2007). Quantitative prediction of cellular metabolism with constraint-based models: The COBRA Toolbox. Nat. Protoc..

[60] Heirendt L., Arreckx S., Pfau T., Mendoza S.N., Richelle A., Heinken A., Haraldsdottir H.S., Keating S.M., Vlasov V., Wachowiak J. (2017). Creation and analysis of biochemical constraint-based models: The COBRA Toolbox v3.0. arXiv.

[61] Schellenberger J., Que R., Fleming R.M., Thiele I., Orth J.D., Feist A.M., Zielinski D.C., Bordbar A., Lewis N.E., Rahmanian S. (2011). Quantitative prediction of cellular metabolism with constraint-based models: The COBRA Toolbox v2.0. Nat. Protoc..

[62] Agren R., Liu L., Shoaie S., Vongsangnak W., Nookaew I., Nielsen J. (2013). The RAVEN toolbox and its use for generating a genome-scale metabolic model for Penicillium chrysogenum. PLoS Comput. Biol..

[63] Arkin A.P., Stevens R.L., Cottingham R.W., Maslov S., Henry C.S., Dehal P., Ware D., Perez F., Harris N.L., Canon S. (2016). The DOE Systems Biology Knowledgebase (KBase). bioRxiv.

[64] Bauer E., Zimmermann J., Baldini F., Thiele I., Kaleta C. (2017). BacArena: Individual-based metabolic modeling of heterogeneous microbes in complex communities. PLoS Comput. Biol..

[65] Harcombe W.R., Riehl W.J., Dukovski I., Granger B.R., Betts A., Lang A.H., Bonilla G., Kar A., Leiby N., Mehta P. (2014). Metabolic resource allocation in individual microbes determines ecosystem interactions and spatial dynamics. Cell Rep..

[66] Louca S., Doebeli M. (2015). Calibration and analysis of genome-based models for microbial ecology. eLife.

[67] Zhuang K., Izallalen M., Mouser P., Richter H., Risso C., Mahadevan R., Lovley D.R. (2011). Genome-scale dynamic modeling of the competition between Rhodoferax and Geobacter in anoxic subsurface environments. ISME J..

[68] Zomorrodi A.R., Islam M.M., Maranas C.D. (2014). d-OptCom: Dynamic multi-level and multi-objective metabolic modeling of microbial communities. ACS Synth. Biol..

[69] Chan S.H.J., Simons M.N., Maranas C.D. (2017). SteadyCom: Predicting microbial abundances while ensuring community stability. PLoS Comput. Biol..

[70] Cottret L., Wildridge D., Vinson F., Barrett M.P., Charles H., Sagot M.F., Jourdan F. (2010). MetExplore: A web server to link metabolomic experiments and genome-scale metabolic networks. Nucleic Acids Res..

[71] Mendes-Soares H., Mundy M., Soares L.M., Chia N. (2016). MMinte: An application for predicting metabolic interactions among the microbial species in a community. BMC Bioinform..

[72] Birkel G.W., Ghosh A., Kumar V.S., Weaver D., Ando D., Backman T.W.H., Arkin A.P., Keasling J.D., Martin H.G. (2017). The JBEI quantitative metabolic modeling library (jQMM): A python library for modeling microbial metabolism. BMC Bioinform..

[73] King Z.A., Lu J., Dräger A., Miller P., Federowicz S., Lerman J.A., Ebrahim A., Palsson B.O., Lewis N.E. (2015). BiGG Models: A platform for integrating, standardizing and sharing genome-scale models. Nucleic Acids Res..

[74] Noronha A., Modamio J., Jarosz Y., Sompairac N., Gonzalez G.P., Danielsdottir A.D., Krecke M., Merten D., Haraldsdottir H.S., Heinken A. (2018). The Virtual Metabolic Human database: Integrating human and gut microbiome metabolism with nutrition and disease. bioRxiv.

[75] Henry C.S., DeJongh M., Best A.A., Frybarger P.M., Linsay B., Stevens R.L. (2010). High-throughput generation, optimization and analysis of genome-scale metabolic models. Nat Biotechnol.

[76] Pornputtapong N., Nookaew I., Nielsen J. (2015). Human metabolic atlas: An online resource for human metabolism. Database.

[77] Kanehisa M., Goto S., Sato Y., Furumichi M., Tanabe M. (2012). KEGG for integration and interpretation of large-scale molecular data sets. Nucleic Acids Res..

[78] Kanehisa M., Goto S., Sato Y., Kawashima M., Furumichi M., Tanabe M. (2013). Data, information, knowledge and principle: Back to metabolism in KEGG. Nucleic Acids Res..

[79] Schomburg I., Jeske L., Ulbrich M., Placzek S., Chang A., Schomburg D. (2017). The BRENDA enzyme information system-From a database to an expert system. J. Biotechnol..

[80] D’Eustachio P. (2011). Reactome knowledgebase of human biological pathways and processes. Methods Mol. Biol..

[81] UniProt Consortium T. (2018). UniProt: The universal protein knowledgebase. Nucleic Acids Res..

[82] Baldini F., Heinken A., Heirendt L., Magnusdottir S., Fleming R.M., Thiele I. (2018). The Microbiome Modeling Toolbox: From microbial interactions to personalized microbial communities. bioRxiv.

[83] Heinken A., Ravcheev D.A., Baldini F., Heirendt L., Fleming R.M., Thiele I. (2017). Personalized modeling of the human gut microbiome reveals distinct bile acid deconjugation and biotransformation potential in healthy and IBD individuals. bioRxiv.

[84] Heinken A., Sahoo S., Fleming R.M., Thiele I. (2013). Systems-level characterization of a host-microbe metabolic symbiosis in the mammalian gut. Gut Microbes.

[85] Gudmundsson S., Thiele I. (2010). Computationally efficient flux variability analysis. BMC Bioinform..

[86] Gorvitovskaia A., Holmes S.P., Huse S.M. (2016). Interpreting Prevotella and Bacteroides as biomarkers of diet and lifestyle. Microbiome.

[87] Biggs M.B., Papin J.A. (2013). Novel multiscale modeling tool applied to Pseudomonas aeruginosa biofilm formation. PLoS ONE.

[88] Weiss S., Van Treuren W., Lozupone C., Faust K., Friedman J., Deng Y., Xia L.C., Xu Z.Z., Ursell L., Alm E.J. (2016). Correlation detection strategies in microbial data sets vary widely in sensitivity and precision. ISME J..

[89] Kurtz Z.D., Muller C.L., Miraldi E.R., Littman D.R., Blaser M.J., Bonneau R.A. (2015). Sparse and compositionally robust inference of microbial ecological networks. PLoS Comput. Biol..

[90] Das P., Ji B., Kovatcheva-Datchary P., Bäckhed F., Nielsen J. (2018). In vitro co-cultures of human gut bacterial species as predicted from co-occurrence network analysis. PLoS ONE.

[91] Duarte N.C., Becker S.A., Jamshidi N., Thiele I., Mo M.L., Vo T.D., Srivas R., Palsson B.O. (2007). Global reconstruction of the human metabolic network based on genomic and bibliomic data. Proc. Natl. Acad. Sci. USA.

[92] Ma H., Sorokin A., Mazein A., Selkov A., Selkov E., Demin O., Goryanin I. (2007). The Edinburgh human metabolic network reconstruction and its functional analysis. Mol. Syst. Biol..

[93] Swainston N., Smallbone K., Hefzi H., Dobson P.D., Brewer J., Hanscho M., Zielinski D.C., Ang K.S., Gardiner N.J., Gutierrez J.M. (2016). Recon 2.2: From reconstruction to model of human metabolism. Metabolomics.

[94] Brunk E., Sahoo S., Zielinski D.C., Altunkaya A., Dräger A., Mih N., Gatto F., Nilsson A., Gonzalez G.A.P., Aurich M.K. (2018). Recon3D enables a three-dimensional view of gene variation in human metabolism. Nat. Biotechnol..

[95] Mardinoglu A., Agren R., Kampf C., Asplund A., Nookaew I., Jacobson P., Walley A.J., Froguel P., Carlsson L.M., Uhlen M. (2013). Integration of clinical data with a genome-scale metabolic model of the human adipocyte. Mol. Syst. Biol..

[96] Mardinoglu A., Agren R., Kampf C., Asplund A., Uhlen M., Nielsen J. (2014). Genome-scale metabolic modelling of hepatocytes reveals serine deficiency in patients with non-alcoholic fatty liver disease. Nat. Commun..

[97] Sahoo S., Thiele I. (2013). Predicting the impact of diet and enzymopathies on human small intestinal epithelial cells. Hum. Mol. Genet..

[98] Sahoo S., Aurich M.K., Jonsson J.J., Thiele I. (2014). Membrane transporters in a human genome-scale metabolic knowledgebase and their implications for disease. Front. Physiol..

[99] Lewis N.E., Schramm G., Bordbar A., Schellenberger J., Andersen M.P., Cheng J.K., Patel N., Yee A., Lewis R.A., Eils R. (2010). Large-scale in silico modeling of metabolic interactions between cell types in the human brain. Nat. Biotechnol..

[100] Väremo L., Scheele C., Broholm C., Mardinoglu A., Kampf C., Asplund A., Nookaew I., Uhlén M., Pedersen B.K., Nielsen J. (2016). Proteome-and Transcriptome-Driven Reconstruction of the Human Myocyte Metabolic Network and Its Use for Identification of Markers for Diabetes. Cell Rep..

[101] Agren R., Bordel S., Mardinoglu A., Pornputtapong N., Nookaew I., Nielsen J. (2012). Reconstruction of genome-scale active metabolic networks for 69 human cell types and 16 cancer types using INIT. PLoS Comput. Biol..

[102] Thiele I., Sahoo S., Heinken A., Heirendt L., Aurich M.K., Noronha A., Fleming R.M. (2018). When metabolism meets physiology: Harvey and Harvetta. bioRxiv.

[103] Roesch L.F., Lorca G.L., Casella G., Giongo A., Naranjo A., Pionzio A.M., Li N., Mai V., Wasserfall C.H., Schatz D. (2009). Culture-independent identification of gut bacteria correlated with the onset of diabetes in a rat model. ISME J..

[104] Wen L., Ley R.E., Volchkov P.Y., Stranges P.B., Avanesyan L., Stonebraker A.C., Hu C., Wong F.S., Szot G.L., Bluestone J.A. (2008). Innate immunity and intestinal microbiota in the development of Type 1 diabetes. Nature.

[105] Brugman S., Klatter F., Visser J., Wildeboer-Veloo A., Harmsen H., Rozing J., Bos N. (2006). Antibiotic treatment partially protects against type 1 diabetes in the Bio-Breeding diabetes-prone rat. Is the gut flora involved in the development of type 1 diabetes?. Diabetologia.

[106] Kostic A.D., Gevers D., Siljander H., Vatanen T., Hyötyläinen T., Hämäläinen A.-M., Peet A., Tillmann V., Pöhö P., Mattila I. (2015). The dynamics of the human infant gut microbiome in development and in progression toward type 1 diabetes. Cell Host Microbe.

[107] Dimeloe S., Burgener A.V., Grahlert J., Hess C. (2017). T-cell metabolism governing activation, proliferation and differentiation; a modular view. Immunology.

[108] Bordbar A., Mo M.L., Nakayasu E.S., Schrimpe-Rutledge A.C., Kim Y.M., Metz T.O., Jones M.B., Frank B.C., Smith R.D., Peterson S.N. (2012). Model-driven multi-omic data analysis elucidates metabolic immunomodulators of macrophage activation. Mol. Syst. Biol..

[109] Segata N., Boernigen D., Tickle T.L., Morgan X.C., Garrett W.S., Huttenhower C. (2013). Computational meta’omics for microbial community studies. Mol. Syst. Biol..

[110] Mardinoglu A., Shoaie S., Bergentall M., Ghaffari P., Zhang C., Larsson E., Bäckhed F., Nielsen J. (2015). The gut microbiota modulates host amino acid and glutathione metabolism in mice. Mol. Syst. Biol..

[111] Lagier J.C., Khelaifia S., Alou M.T., Ndongo S., Dione N., Hugon P., Caputo A., Cadoret F., Traore S.I., Seck E.H. (2016). Culture of previously uncultured members of the human gut microbiota by culturomics. Nat. Microbiol..

[112] Lagier J.-C., Hugon P., Khelaifia S., Fournier P.-E., La Scola B., Raoult D. (2015). The rebirth of culture in microbiology through the example of culturomics to study human gut microbiota. Clin. Microbiol. Rev..

[113] David L.A. (2018). Toward Personalized Control of Human Gut Bacterial Communities. mSystems.

[114] Thiele I., Clancy C.M., Heinken A., Fleming R.M. (2017). Quantitative systems pharmacology and the personalized drug–microbiota–diet axis. Curr. Opin. Syst. Biol..

[115] Sen P., Vial H.J., Radulescu O. (2016). Mathematical modeling and omic data integration to understand dynamic adaptation of Apicomplexan parasites and identify pharmaceutical targets. Compr. Anal. Parasite Biol. Metab. Drug Discov..

[116] Sen P., Vial H.J., Radulescu O. (2013). Kinetic modelling of phospholipid synthesis in Plasmodium knowlesi unravels crucial steps and relative importance of multiple pathways. BMC Syst. Biol..

